# Cancer Stem Cells and Immunosuppressive Microenvironment in Glioma

**DOI:** 10.3389/fimmu.2018.02924

**Published:** 2018-12-21

**Authors:** Qianquan Ma, Wenyong Long, Changsheng Xing, Junjun Chu, Mei Luo, Helen Y. Wang, Qing Liu, Rong-Fu Wang

**Affiliations:** ^1^Department of Neurosurgery in Xiangya Hospital, Central South University, Changsha, China; ^2^Center for Inflammation and Epigenetics, Houston Methodist Research Institute, Houston, TX, United States; ^3^Institute of Biosciences and Technology, College of Medicine, Texas A&M University, Houston, TX, United States; ^4^Department of Microbiology and Immunology, Weill Cornell Medical College, Cornell University, New York, NY, United States

**Keywords:** glioma, cancer stem cell, tumor microenvironment, immunosuppression, immunotherapy

## Abstract

Glioma is one of the most common malignant tumors of the central nervous system and is characterized by extensive infiltrative growth, neovascularization, and resistance to various combined therapies. In addition to heterogenous populations of tumor cells, the glioma stem cells (GSCs) and other nontumor cells present in the glioma microenvironment serve as critical regulators of tumor progression and recurrence. In this review, we discuss the role of several resident or peripheral factors with distinct tumor-promoting features and their dynamic interactions in the development of glioma. Localized antitumor factors could be silenced or even converted to suppressive phenotypes, due to stemness-related cell reprogramming and immunosuppressive mediators in glioma-derived microenvironment. Furthermore, we summarize the latest knowledge on GSCs and key microenvironment components, and discuss the emerging immunotherapeutic strategies to cure this disease.

## Introduction

Glioma is one of the most common primary tumors of the central nervous system (CNS). According to the criteria established by the World Health Organization (WHO) and based on various histopathological characteristics and prognostic factors, gliomas can be classified into four different grades (I–IV). Grade I includes pilocytic astrocytoma; diffuse or anaplastic astrocytomas and oligodendrogliomas are categorized as grades II–III and glioblastomas (GBMs) are categorized as the most malignant grade (grade IV) (1, 2). Despite of advanced multimodal therapeutic strategies, which combine aggressive surgery, radiation, and chemotherapy, patients with GBM have dismal prognosis, with median overall survival time of < 16 months (3). Resistance of malignant gliomas to conventional therapies has been widely reported as a consequence of oncogenic signaling activation and distinct metabolic mechanisms when cancer cells are exposed to various chemotherapeutic and/or cytostatic agents (4–7). Furthermore, infiltration of immune cells (with critical activities) into tumor regions contributes to the establishment of an immunosuppressive tumor microenvironment (TME) to promote tumor development, metastasis, and resistance to cancer therapies (8, 9).

In this review, we will discuss the role of key subpopulations of stem cells and their orchestrated immunological interactions with TME in gliomas. In addition, we will further delineate the current immunotherapeutic approaches for targeting the rare subpopulation of stem cells. Comprehensive understanding of the complex crosstalks among these cells and processes will facilitate the establishment of therapeutic strategies, which might eventually cure this malignant disease.

## Glioma Stem Cells (GSCs)

The stem cell theory for cancer maintenance and recurrence has been proposed many years ago and the premise of this theory is consistently being supported by studies conducted in hematopoietic malignancies and solid tumors (10). The tumors are composed of small and rare subpopulation of cells with stem-like properties such as self-renewal, multi-lineage differentiation potential and resistance to conventional treatments. In malignant gliomas, similar to other types of cancer, several studies have identified and isolated the GSCs that harbor tumor-initiating properties (11, 12). Although the origin of these multipotent cells is not clearly defined, the GSCs are thought to reside at the apex of hierarchy in tumorigenesis with potential to induce angiogenesis, metastasis and modulate therapeutic responses. Further, due to an enhanced DNA repair capacity, GSCs recover rapidly from conventional therapeutic stress, which leads to resistance and eventual disease relapse in glioma patients (13). Since GSCs play critical role in tumorigenesis of glioma, great strides have been made to discover unique cellular characteristics and genetic pathways of these cells.

Cell surface molecules differentially expressed on GSCs and functionally associated with the maintenance of GSCs are ideal markers for sorting and targeting GSC population. The classic biomarker used to define GSCs is CD133. CD133^+^ subpopulation of glioma cells exhibit increased self-renewal and proliferation properties *in vitro* and these cells are capable of initiating tumor within brain which retains homogeneous histological features of the original donor (12). In addition, GSCs also exhibit CD15, CD36, CD44, and CD49f/Integrin markers, indicating that there is a possibility of targeting GSCs through specific monoclonal antibodies against these surface markers. However, these surface markers are also expressed on normal neural stem cells (NSCs). Further, to make matters worse, the definition of surface markers of GSCs has been challenging despite of the functional evidence for its stem-like behavior in certain cell subpopulations of gliomas. For example, some notable CD133^−^ glioma cells have been reported as extremely malignant phenotype with stronger tumor-promoting potentialities (14, 15). Increasing evidence suggests that a number of crucial signal transduction pathways are involved in the maintenance of GSCs. Most notable ones are Notch, Sonic Hedgehog, Wnt/β-catenin, Akt, and STAT3 signaling pathways. However, it will be difficult to target these pathways since there is considerable overlap between NSCs and GSCs.

**Figure 1:**
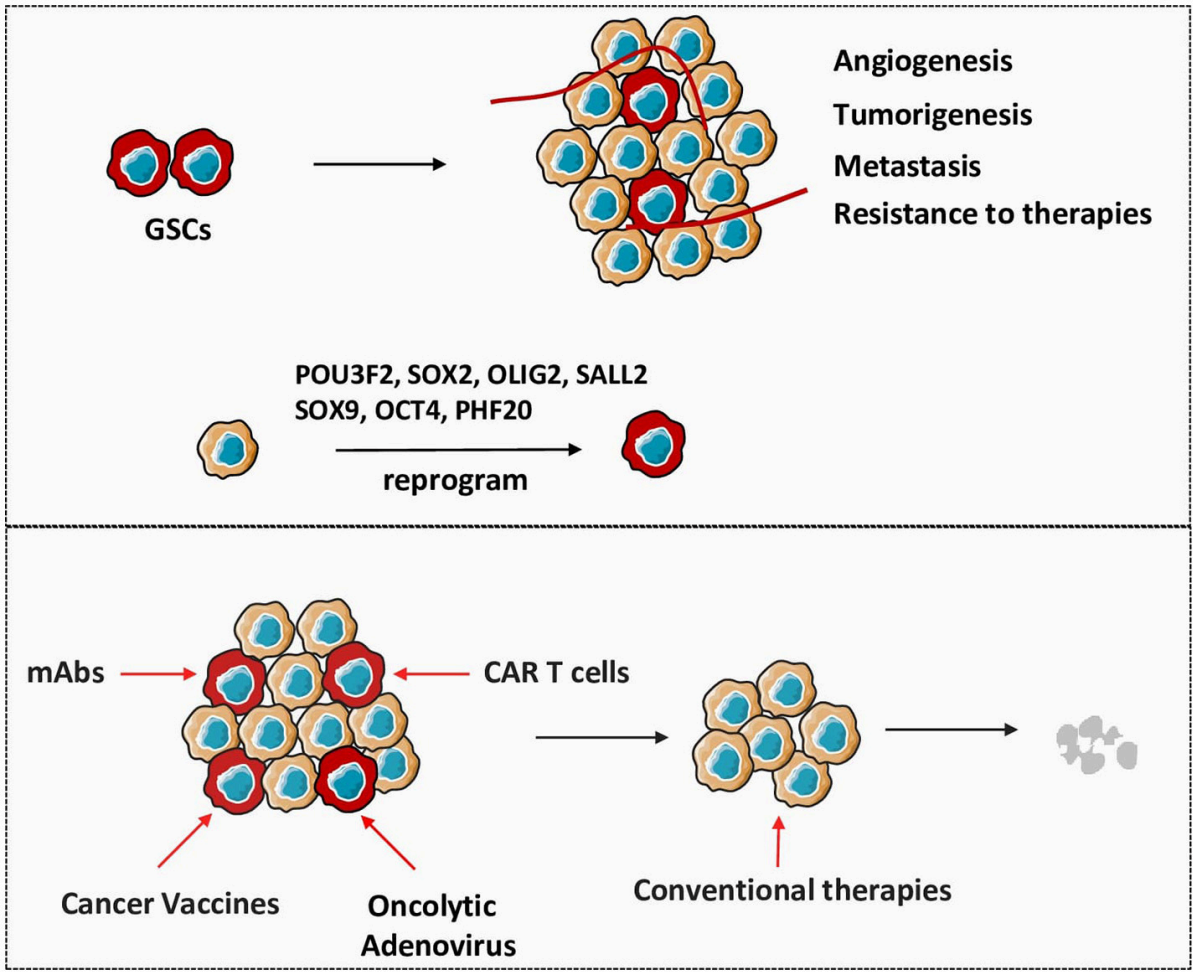
Therapeutic approaches targeting GSCs are critical in glioma treatment. GSCs play important roles in the establishment and recurrence of glioma. Non-stem glioma cells are capable to reprogram to GSCs under the influence of crucial stem genes. Directly targeting GSCs by different strategies will be efficient to gradually eliminate tumor in combination with conventional therapies.

It is well established that cellular reprogramming can convert differentiated somatic cells into inducible pluripotent stem cells (iPSCs) by enforced expression of four factors: SOX2, OCT4, KLF4, and c-MYC (16, 17). Inspired by iPSCs technology and the similarity between iPSCs and cancer stem cells reprogramming, researchers generated glioma stem-like state cells through a dedifferentiated process of glioma cells by overexpression of crucial genes: POU3F2, SOX2, OLIG2, and SALL2 (18), which indicates the impact of critical tumor-promoting genes on the fate of GSCs and further regulation of glioma development. Thus, many transcriptional factors with well-recognized functions in embryonic development have subsequently been identified as oncogenic drivers in tumors, including PHF20, SOX2, SOX9, and OCT4. Notably, PHF20 was initially discovered as a tumor specific antigen in GBM. Patients treated with PHF20 antibody have significantly better outcomes than those without antibody treatment (19). Our previous study showed that PHF20-deficient mouse embryonic fibroblasts could not be converted to fully reprogrammed iPSCs by down regulating OCT4, which revealed that this protein exerts predominant effects on reprogramming (17). Subsequently, PHF20 was found abundantly expressed in neurogenic tumors and plays a vital role in carcinogenesis by significantly up-regulating the expression of SOX2 and OCT4, further enhancing the self-renewal and tumor-initiating capability of neuroblastoma (20). Noteworthy, previous studies have shown high expression of SOX2 and SOX9 in GSCs subpopulation and that these proteins are important for GSC maintenance (21, 22). In addition, recent studies including our ongoing experiments, suggest that deletion of SOX2, SOX9, and OCT4 impair GSCs activities and delay the onset of tumorigenesis (23, 24)_ENREF_35. Collectively, these studies demonstrate the pivotal role of PHF20-SOX2-SOX9-OCT4 axis in aggressive behavior of GSCs (Figure 1). Moreover, interrogating the interactions of these specific stem genes in different contexts may shed some light on establishing the origin of gliomas and provide us with novel therapeutic options to target GSCs.

## Immunotherapeutic Strategies Targeting GSCs (Figure 1)

### Monoclonal Antibodies (mAbs)

The use of antibodies for treating patients with cancer has been established for 20 years and mAbs are one of the major contributions of tumor immuno-oncology with their potential to induce direct cell killing and regulate cellular immune response (25). Given the various markers define GSCs, the mAb therapy proposes one of the most promising approaches to target this malignancy. Amplification and mutation of the epidermal growth factor receptor (EGFR) represents crucial genetic signature in GSCs and mAbs directly targeting EGFR is used as a well-known therapeutic approach in glioma. Cetuximab, the most notable mAb against EFGR, functionally prevents EGFR-mediated signaling by interfering with ligand binding and EGFR extracellular dimerization. In addition, cetuximab might also trigger EGFR receptor internalization and destruction (26). Other unconjugated mAbs against EGFR, such as panitumumab and nimotuzumab, exhibit similar efficacy against GSCs as cetuximab (27). The autocrine TGF-β signaling is involved in multiple cellular processes in tumor development and high serum levels of TGF-β are detected in malignant glioma which positively correlated with tumor grade and prognosis. Additionally, the TGF-β signaling has been reported as a key regulator in the maintenance of GSCs (28). Studies have shown that the activation of TGF-β related pathways induce self-renewal and inhibition of differentiation in GSCs through the regulation of various stem genes, such as SOX4, SOX2, and LIF (28, 29). Numbers of TGF-β targeting therapies are currently under investigation in diverse studies, showing safety and effective outcome for glioma patients. The TGF-β neutralizing antibody, GC1008, shows significant improvement in the survival of patients with recurrent gliomas, with no major toxicity observed during treatment (30). Another important mAb for glioma treatment is bevacizumab, a FDA-approved humanized mAb against VEGF. A number of clinical trials are currently evaluating efficacy of bevacizumab in combination with other therapeutic approaches in patients with newly diagnosed glioma (31). Although bevacizumab is known to have outstanding anti-angiogenic property, one potential mechanism by which bevacizumab inhibits glioma growth is due to disturbance of the perivascular niche where GSCs reside. Disruption of the supportive microenvironment causes GSCs more susceptible to damage from other therapies (31, 32).

### Cancer Vaccines

In contrast to mAbs, cancer vaccines are classified into a subcategory of active immunotherapy due to their ability to motivate the host immune system to recognize and kill tumor cells. The most successful access of vaccine is the ability to harness the potent antigen-presenting processes of dendritic cells (DCs). Every component from tumor cells can be utilized in stimulation of DCs, leading to their recognition of tumor-associated antigens (TAAs). After reintroduction into patients, the stimulated DCs mediate prolonged antitumor response by presenting TAAs to tumor specific T cells (33). In our previous study, we report the co-delivery of tumor specific antigen and dual toll-like receptors (TLRs) into DCs via a novel microparticle (mesoporous silicon vector) induce efficient host immune responses against melanoma (34). Interestingly, as cellular components from killed tumor cells boost DC function for active immunostimulation, the efficacy and safety of postoperative administration of autologous DC vaccination is evaluated in a clinical trial. These clinical studies show that lysate-pulsed DC vaccination is efficient and safe following tumor resection (35). Given the identical biological properties and tumorigenic potential of GSCs, it is possible to create comprehensive immune therapies against glioma by directly targeting this subpopulation of tumor cells (36). In the first GSCs targeted vaccine therapy in humans, single GSCs are separated and allowed to form tumorsphere, and the mRNA isolated from autologous GSCs are transfected into monocyte-derived DCs. Patients that received this vaccine treatment via intradermal injection following synergic anti-tumor therapy show promising clinical outcomes without serious adverse reactions (37). In one clinical trial, a variety of TAAs which are specifically associated with tumorigenic GSCs (HER-2, AIM-2, gp100, IL13Rα2, TRP-2, MAGE1) are targeted by ICT-07 vaccine and the results demonstrate improvement of the progression-free survival. Intriguingly, in those patients who require double surgery, the second tumor sample exhibits decreased number of GSCs, indicating vaccine efficacy in targeting these cells within tumor (38). As the CNS largely lacks activated DCs in the processes of active immunotherapy, most currently, hematopoietic stem and progenitor cells (HSPCs) have been administrated intracranially to supply resident DCs and to induce effective T cell responses. When transferring into CNS, HPSCs differentiate into CD86^+^CD11c^+^MHCII^+^ cells which present activated DCs phenotype and function to promote cytotoxic immune responses. The HSPC-derived cells are extremely effective due to the high expression of co-stimulatory markers like CD86, which facilitates potent anti-tumor immunity (39). In recent years, SOX2, a transcription factor which plays an essential role in the maintenance of GSCs, has been reported to represent a novel target for active immunotherapy. Vaccinations with SOX2 peptides significantly enhance systemic and local immune response and prolong overall survival in animal models in combination with or without chemotherapy (40). In this regard, we assume that targeting SOX2, likely together with selected oncogenes, may introduce us to a new approach to treat GSCs through active immunotherapies.

### Chimeric Antigen Receptor (CAR) T Cells

Distinct to active immunotherapies which stimulate the innate immune system by TAAs, the adoptively transferred CAR T cells directly target tumor antigens independent of antigen presentation to perform their anti-tumor activities. CAR, which links TAA-specific mAb to T cell activation signaling, are designed to target any antigen on surface of tumor cells and transferred to T cell populations (41). Given that intrinsic tumor specific T cells are infrequent and anergic, the CAR T cells could be expanded *in vitro* to sufficient levels to induce potent cytotoxic immune responses. Despite the successful application of CAR T cells in hematologic malignancies, this therapeutic strategy is not well-developed in solid tumors due to the deficiency of antigens that are exclusively expressed on tumor surface and the involvement of the suppressive microenvironment in the setting of tumor bed (42). Considering the strong side effects of current CAR T therapies in CNS like hydrocephalus, the use of CAR T cells is even rare in the treatment of glioma (43, 44).

However, a tumor specific mutation of EGFR, the EGFRvIII, which is most frequently seen in patients with glioma, has emerged as an ideal target for CAR T cell treatment. The CART-EGFRvIII cells have been found effective and safe in patients with residual or recurrent glioma (45). Besides EGFRvIII, disialoganglioside GD2 is observed in high levels in patients with diffuse intrinsic pontine glioma, and the GD2 targeted CAR T cells demonstrate potent antitumor efficacy and well controlled T cell-related inflammation (46). Number of preclinical studies have revealed that IL13Rα2 and HER2 can be targeted and ablated with administration of CAR T cells in GSCs (47, 48). These studies indicate that GSCs can be potentially targeted with appropriately designed CAR T cells.

### Oncolytic Adenovirus

Oncolytic adenoviruses have been documented as promising anti-tumor agents based on their abilities to selectively replicate in and effectively kill cancer cells, including cancer stem cells (49). Besides the classical cancer-specific replication and lytic activity, adenoviral infection also modulates host immunity which presents a more comprehensive understanding of oncolytic virus-associated anti-cancer activities (50). The use of oncolytic adenoviruses as a potent therapeutic approach against GSCs have been explored extensively (51, 52). In a phase I clinical trial, DNX-2401 (Delta-24-RGD; tasadenoturev), a tumor selective and infectivity enhanced oncolytic adenovirus is injected into patients with recurrent malignant glioma. The phase I study (administration of DNX-2401) demonstrates a good safety record, dramatic reduction in the tumor size and long-term survival in this cohort of patients. In addition to direct oncolytic effect, elevated immune cell infiltration and enhanced cytotoxic T cell function also participate in the effective antitumor responses thereby, indicating significant clinical efficacy of virotherapy (52). Ongoing clinical trial (NCT02798406) focused on the combination therapy with DNX-2401 and PD-1 inhibition should provide more promising evidence pertaining to oncolytic adenovirus-based anti-glioma strategies.

### Glioma-Associated Microenvironment

**Figure 2:**
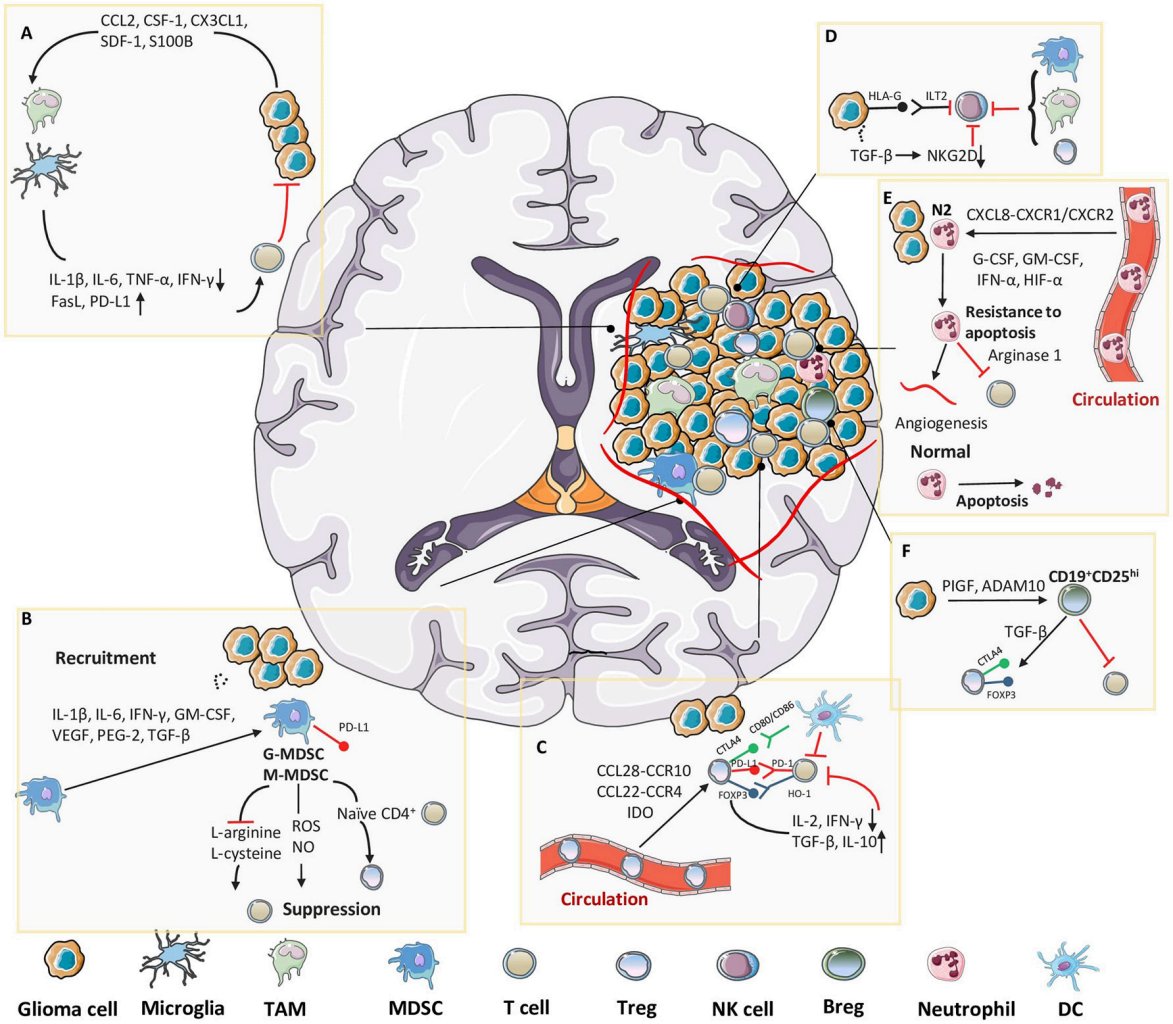
Immunosuppressive cellular components in glioma microenvironment. Tumor cells release molecules which contribute to multiple unique immunosuppression mediated by various cellular players in glioma microenvironment. **(A)** Cytokines or chemoattractants secreted by glioma cells induce peripheral-derived TAMs and resident microglia to possess M2 phenotype, which enables the production of tumor-promoting factors. **(B)** Tumor cells induce massive infiltration of MDSCs via multiple approaches, which further exert an immunosuppressive function mainly through T cell inhibition. **(C)** After recruited to the tumor site, Tregs directly suppress the activity of cytolytic T cells and induce their apoptosis. **(D)** Despite well-characterized specialties in recognizing and killing tumor cells, the functions of NK cells in glioma microenvironment are limited due to the existence of HLA-G or TGF-β. TAMs, MDSCs and Tregs also collaborate to suppress the activity of NK cells. **(E)** Tumor-associated neutrophils (TANs) are attracted into tumor bed, where they could evade apoptosis by interacting with diverse molecules, and further benefit the inner-tumor angiogenesis and T cell suppression. **(F)** In glioma microenvironment, PIGF and ADAM10 cooperate to induce suppressive CD19^+^CD25^hi^ Bregs, which are responsible for Tregs proliferation and suppression of other T cells.

Human gliomas produce numerous cytokines, chemokines, and growth factors, which in turn promote the infiltration of various immune cells into tumors^14, 15^. Although CNS is “immune privileged,” the brain blood barrier (BBB) has been shown to be compromised (the exchange of many substances between the brain and blood circulation) under specific pathological conditions (7, 53). In patients with high-grade glioma, the BBB appears to lose its effective function due to abnormal sprouting structures, tumor-induced formation of new vessels, and dysfunction of tight junctions, leading to severe infiltration of inflammatory cells (54). Additionally, peripheral antitumor immune cells are frequently reprogrammed into a distinct immunosuppressive phenotype in TME soon after their chemotaxis to the tumor (4, 55). Various types of immune cells in solid glioma tissue directly regulate the interactions between the tumor and host, which can help the tumor suppress, modulate, or even evade immune responses at different levels (6, 53) (Figure 2).

### Tumor-Associated Macrophages (TAMs) and Microglia

Clinical studies have shown that malignant gliomas are extensively infiltrated by myeloid-derived cells, including TAMs and microglia. Most TAMs arise from circulating monocytes, which are recruited to the brain parenchyma under pathological conditions (56, 57). This is relevant in brain tumors, particularly glioma, which is always accompanied by disruption of BBB during disease progression (58). Brain microglia arise from embryonic yolk sac progenitor cells, and unlike other tissue-resident macrophages, this unique population acquires self-renewal ability. Moreover, microglia also exhibit prolonged cellular longevity and local proliferation within the brain (57). Because of their considerable diversity and plasticity, TAM populations can be divided into two groups, the “classically activated” M1 phenotype and the “alternatively activated” M2 phenotype (59, 60). Malignant gliomas actively recruit microglia and macrophages to tumor sites where these cell acquire amoeboid morphology and adopt an M2 tumor-promoting phenotype, thus contributing to the immunosuppressive tumor environment (58). This recruitment is mediated through the secretion of various chemo-attractants, such as C-C motif chemokine ligand (CCL) 2, colony-stimulating factor (CSF)-1, CX3CL1, and stromal-derived factor (SDF)-1. CCL2, also known as monocyte chemotactic protein-1, is first identified in glioma cells as a cytokine that could induce the accumulation of TAMs around tumor tissues (61). Ectopic expressions of CCL2 both at the mRNA and protein levels are strongly correlated with enhanced infiltration of TAMs at tumor sites and promote tumor aggressiveness in an experimental animal model (62). Moreover, overexpression of CCL2 is induced by upregulation of protein S100 calcium binding protein B, and this positive correlation between the two proteins promotes TAM recruitment in the glioma microenvironment (63). Macrophages also critically depend on the multiple functions of CSF-1, which is constitutively expressed by glioma cells, facilitated by its receptor CSF-1R. Attenuated interactions between CSF-1 and CSF-1R with CSF-1R-targeting inhibitors reduce the number of TAMs in tumor sites and impair the invasion ability of glioma cells.

After migrating into the glioma environment, TAMs tend to possess the M2 phenotype, and their differentiation is driven by the secretion of immunosuppressive factors, including CSF-1, CCL2, IL-4, IL-6, IL-10, and transforming growth factor (TGF- β), from tumor cells (64, 65). Additionally, ectopic activation of the signal transducer and activator of transcription (STAT) 3 pathway has been reported to play a crucial role in the maintenance of the M2 phenotype of TAMs (66). Vascular cell adhesion protein-1 expression mediated by STAT3 is positively correlated with TAM adhesion and immunosuppressive function in gliomas (67). The switch of glioma-associated TAMs in turn leads to the establishment of an immunosuppressive microenvironment via direct impairment of T-cell activation and proliferation due to the lack of expression of costimulatory molecules (CD40, CD80, and CD86) and the production of low levels of pro-inflammatory cytokines (IL-1β, IL-6 and TNF-α) upon TLR stimulation, thus contributes to the failure of T-cell stimulation and proliferation and makes T-cells less capable of mediating tumor cytotoxicity compared with microglia isolated from the normal brain (68, 69). In contrast, overexpression of immunosuppressive mediators, such as FasL and programmed cell death-ligand 1 (PD-L1) on the surface of TAMs in glioma microenvironment also result in the apoptosis of T cells and deficiencies in immunosurveillance (70). When M2 TAMs release low levels of IFN-γ and high levels of IL-10, microglia act as potent regulatory T cells (Tregs) inducer and further supports immune suppression in the glioma environment (71). M2 TAMs may also help to induce the immunosuppressive niche by decreasing lymphokine-activated killer cells, natural killer (NK) cells, and cytotoxic T lymphocyte (CTL) activity. TAMs consistently produced anti-inflammatory cytokines, such as IL-10, TGF-β, and IL-6, thereby mediating a wide range of immunosuppressive functions (72, 73).

### Myeloid-Derived Suppressor Cells (MDSCs)

MDSCs are heterogeneous population of immature myeloid cells consisting of myeloid progenitors and precursors of macrophages, granulocytes, and dendritic cells (74, 75). MDSCs share some common features such as their myeloid origin, immature state, and most importantly, the ability to convert immune responses from a Th1 phenotype toward a Th2 phenotype, which result in potent inhibition of CD4^+^ and CD8^+^ T cells and significant immunosuppression in tumor settings (76, 77). Direct interaction between glioma cells and monocytes is required to achieve a tumor-promoting immunosuppressive phenotype. And extensive MDSC infiltration around the TME has been observed in all glioma models and patients. Glioma cells utilize several approaches to induce the undifferentiated state and stimulate the expansion of MDSCs. Multiple chemokines, e.g., pro-inflammatory factors (IL-1β, IL-6), activated T cell-derived cytokines (IFN-γ, IL-4, IL-10, and IL-13) and multiple soluble mediators secreted by gliomas (granulocyte macrophage [GM]-CSF, vascular endothelial growth factor [VEGF], PGE-2, and TGF-β2), attract MDSCs toward the tumor and synergistically initiate immunosuppressive pathways that commit immature myeloid cells to become MDSCs; this then further promotes the differentiation of MDSCs toward TAMs (78–80).

After recruitment to the tumor site, MDSCs exhibit four distinct T-cell inhibitory effects. The first is the direct depletion of nutrients essential for the growth and differentiation of lymphocytes, such as L-arginine and L-cysteine. Further, proliferation of antigen-activated T-cells is blocked due to decrease in T-cell receptor-associated CD3ζ chain, which is caused by lacking of amino acids, L-arginine and L-cysteine. Consistent with this notion, arginine supplementation can relieve T-cell suppression and restore T-cell function in gliomas. The second effect is the interaction with naïve CD4^+^ T cells; upregulation of PD-L1 on the surface of tumor-derived MDSCs leads to the functional exhaustion of CD4^+^ effector T cells. Moreover, MDSCs can also mediate the conversion of naïve CD4^+^ T cells into induced Tregs by producing cytokines. TGF-β produced by tumor-associated MDSCs, together with IL-10 and scarcity of arginine expression, has been shown to facilitate the differentiation and expansion of forkhead box P3 (FOXP3)^+^ Tregs in other tumor types. Based on the overexpression of these factors and the existence of both MDSCs and Tregs in the glioma microenvironment, researchers hypothesize that MDSC-mediated expansion of FOXP3^+^ Tregs may also occur in gliomas (81). The third type of mechanism is the generation of reactive nitrogen species and reactive oxygen species (ROS). Nitric oxide (NO) production in animal models of glioma has been documented as a potent inducer of T-cell suppression through induction of oxidative stress. Finally, the fourth effect deals with impairment of T-cell trafficking and viability (77, 82). Moreover, increased levels of IL-10 and PD-L1 observed on the surface of MDSCs during coculture with glioma cells indicates that MDSCs may induce T-cell anergy through binding with PD-1 (83, 84). Since both MDSCs and TAMs around the glioma microenvironment express high levels of IL-10 and are skewed toward an immunosuppressive phenotype by IL-10, it is likely that MDSCs and TAMs promote polarization of each other in glioma. Thus, coordinated regulation between PD-L1 and IL-10 may result in reduced glioma-derived antigen presentation and hamper the effective antitumor response even further.

### Tregs

High occurrences of Tregs are found in tumor sites of various types of cancers, and studies have shown that hyperactivity of Treg function may be associated with tumor evasion (85, 86)_ENREF_84. We have identified tumor-specific Tregs in several types of cancers (87, 88)_ENREF_85. In particular, we demonstrate that the suppressive function of human naturally occurring CD4+ CD25+ Treg cells and tumor-derived antigen-specific CD4+ Treg cells can be reversed by TLR8 signaling pathway in DCs independent mechanism, but requires the activation of TLR8-MyD88-IRAK4 signaling pathway (89). Further, Tregs are reported to have keys role in the pathogenesis of glioma (90). For instance, glioma patients show significant infiltration of Tregs that correlates with WHO grades. Studies have shown that GBM has the largest amount of Tregs around the tumor tissue, further indicating a direct correlation between tumor malignancy and recruitment/infiltration of Tregs (91). The organized combination of several chemokines secreted by tumor cells and the expression of chemokine receptors on the surface of Tregs lead to the expansion and migration of tumor-associated Tregs from circulation. For example, tumor-derived Tregs are continuously induced via the CCL22/CCR4 and CCL28/CCR10 axes in GBM multiforme. Further, Treg recruitment is secondary to the release of these chemoattractants, implying that although Tregs may be necessary for tumor development, they are not contributing the initial establishment of the tumor (92, 93). Additionally, in GBM animal models, indoleamine 2,3-dioxygenase (IDO), which is expressed on tumor cells, was found to stimulate recruitment of Tregs, resulting in the impairment of immune surveillance (94).

After infiltrating into tumor sites, Tregs are activated through recognition of tumor-specific antigens and self-antigens (released from dying tumor cells) which positively regulate the immunosuppressive TME by attenuating tumor-specific effector T cells. In absence of Tregs, CD8^+^ T cells from glioma patients are able to restore their proliferative and cytotoxic activities, while CD4^+^ T cells were able to expand in response to antigen stimulation (95). The suppressive role of Tregs in the antigen-presenting process is the primary pattern of Treg-mediated immunological self-tolerance. Antigen-presenting cells, such as lymphocytes and dendritic cells (DCs), are inhibited by Tregs through downregulation of IL-2 and IFN-γ, which are crucial factors responsible for activation of both T cells and DCs (90). Additionally, immunosuppressive mediators, such as TGF-β and IL-10, are secreted from target cells which further enhance the induction of Tregs (96). Furthermore, FOXP3, a unique marker expressed on the surface of Tregs, has also shown to contribute to immunosuppression. Specifically, FOXP3 induces the expression of heme oxygenase-1, which engages in FOXP3-mediated immune suppression in a cell-to-cell contact-dependent manner, further impairing the proliferation of T cells (90, 97). Another possible mechanism for suppressing the function of Tregs is mediated through immune-checkpoint molecules constitutively expressed on the surface of Tregs, e.g., CTLA-4 and PD-L1 (98–100). Ectopic expression of these molecules and their ligands (CD80, CD86, and PD-L1) downregulates the activities of antigen-presenting cells and tumor-specific T cells through direct cell-to-cell interactions with Tregs (101).

### NK Cells

NK cells are an important type of cytotoxic innate lymphocytes that provide rapid responses to viral infections and tumor formation. In the presence of surface activating receptors and absence of inhibiting receptors, NK cells are able to directly lyse MHC-I-deficient tumor cells or other pathogens (102). Under normal circumstances, activation of NK cells is inhibited by binding surface inhibitory receptors with HLA class I antigens to protect nonpathological cells from being killed and to maintain homeostasis. Disruption of homeostasis by various pathological changes reduces HLA-I expression and impairs the immune-tolerance mediated by NK cells (103). However, ectopic high expression of HLA-I found in malignant gliomas through different tumor-dependent mechanisms can further strengthen the impairment of immune surveillance (104). HLA-G, an inhibitory ligand in GBM, is found to bind to its receptor Ig-like transcript 2 on NK cells, activating a major tumor-immune escape mechanism (105, 106). Moreover, epigenetic signals or cytokines, such as TGF-β, secreted locally by glioma cells can dysregulate the expression of natural-killer group 2, member D, an activator of NK cells, thereby inhibiting tumor-specific cytolysis and preventing the immune response (107, 108). Additionally, direct contacts with other immunosuppressive cells around the glioma, such as glioma-associated macrophages, MDSCs, and Tregs, also induce the infiltrated NK cells to exhibit a nonfunctional phenotype (72, 109, 110).

### Neutrophils

Neutrophils are the most abundant granulocytes in humans, comprising nearly 70% of all leukocytes in the body, and are considered as the first line of defense in the innate immune response under pathological conditions (111). Similar to macrophages and MDSCs, tumor-associated neutrophils (TANs) may also participate in immune suppression and subsequent tumorigenesis and tumor growth (112, 113). The phenotype of TANs can be divided into two distinct subsets (N1 and N2), with the anti-tumor N1 type modulating IFN-γ while the tumor-promoting N2 type regulating TGF-β; the N2 type is the major subtype observed in the TME (114). Elevated numbers of neutrophils have been found to be correlated with poor patient survival in severaltypes of cancer (115, 116), including glioma (117), suggesting that the infiltration of neutrophils may affect tumor immunosuppression. Several studies have shown that chemo attractants released by tumor cells induce the recruitment and infiltration of neutrophils into tumor sites and subsequently tilt them to N2 TANs. As the most potent neutrophil-attracting mediator, CXCL8 plays a critical role in orchestrating neutrophil recruitment by binding to the receptors CXCR1 and CXCR2 (118). In glioma, high level of CXCL8 is found in tumor cells, and correlated with increased neutrophil infiltration and tumor progression (119). Since neutrophils are very short-lived cells with a circulating half-life of < 1 day, the apoptotic process is strongly inhibited in these cells by some specific prosurvival factors (e.g., G-CSF, GM-CSF, and IFN-α) (115), as well as activated CD4^+^ and CD8^+^ T lymphocytes (120). Furthermore, the hypoxic environment facilitates resistance of neutrophils to apoptosis in a hypoxia-inducible factor (HIF)-1α-dependent manner (121). Stimulation with these factors promotes the survival and immunosuppressive functions of neutrophils to enhance tumor progression.

Most studies of neutrophils and gliomas have demonstrated the effect of these cells in response to tumor angiogenesis and anti-angiogenic therapies. Neutrophils are able to influence angiogenesis inside tumors by releasing angiogenic factors, which promote endothelial cell migration and proliferation (116). Further, neutrophils may also promote angiogenesis by shifting GSCs from a proneural stage to mesenchymal subtype through upregulation of S100A4 (122). Moreover, neutrophils may also play role in the immunosuppression of glioma TME. Arginase 1 is a critical immunosuppressive molecule that can directly inhibit CD8 T-cell function and stimulate TAMs to promote tumor growth (123). Neutrophils are stimulated to release immunosuppressive arginase 1 by supernatants from tumor cells in a CXCL8-dependent manner, and knockdown of CXCL8 blocks this effect (124). In addition, elevation of neutrophil-to-lymphocyte ratio and increased intratumoral infiltration of neutrophils are documented to be correlated with a decreased CD3^+^ T-cell infiltration in tumor samples from patients with glioma (117). Mutation of isocitrate dehydrogenase 1 results in improved clinical outcomes and has been shown to decrease leukocyte chemotaxis and immune infiltration of TANs, thereby blocking malignant immunosuppression in glioma (125).

### B Cells and Regulatory B Cells (Bregs)

In addition to the contribution of TAMs, MDSCs, Tregs, and TANs in suppression of the antitumor immune response, subsets of B cells with immunosuppressive or regulatory abilities have recently emerged as contributors to immune responses in the pathogenesis of autoimmune diseases and human tumors (126, 127). Bregs are originally identified in autoimmune disease; however, recent studies have shown that interaction between Bregs and many other immune cell types in the TME augment the suppressive microenvironment and promote tumor growth (128). Various human solid tumors exhibit upregulation of tumor-infiltrative Bregs as malignant B cells that suppress the antitumor cellular immune response through the expression of suppressive ligands (129–131). Additionally, human CD19^+^CD25^hi^ B cells (which suppress CD4^+^ T-cell proliferation and enhance CTLA-4 and FOXP3 expression on the surface of Tregs in a TGF-β-dependent manner) are considered representatives of the Breg population (132). Further, CD19^+^CD24^hi^CD38^hi^ B cells in the peripheral blood circulatory system are responsible for suppressing the production of IFN-γ and TNF-α from CD4^+^ T cells (133).

It has been shown in many different tumors that secretion of various cytokines (including IL-6, IL-10, IL-35, and TGF-β) from Bregs support the activation and expansion of Tregs, TAMs, and MDSCs around the tumor bed, thereby skewing these cells toward suppressive subsets to enhance the tumor-promoting microenvironment (134, 135). In addition, placental growth factor (a member in the VEGF family), secreted by glioma tumor cells appears to switch tumor-infiltrating B cells to Bregsand suppress the CD8^+^ T-cell response (136). Similarly, glioma tumor cells can also induce Bregs by releasing the disintegrin and metalloproteinase domain-containing protein 10 and contribute to the recruitment of Tregs and suppression of CD8^+^ T cells (137).

### Hypoxia and Immunosuppression of the TME

Hypoxia is a hallmark of all solid tumors and is the consequence of abnormal morphology of blood vessels, which are unable to deliver adequate oxygen and nutrients to rapidly proliferating tumor cells (138). This pathological condition contributes to a number of events, including angiogenesis, effective T-cell suppression, and neoplastic progression (139). Consequently, the TME also evolves a mechanical switch to counterbalance the low level of oxygen and support the acquisition of properties in tumor cells required to adapt to the hypoxic surroundings. This adaptive transcriptional program is mainly mediated by HIF proteins (140). Hypoxic conditions dominate most TMEs; thus, HIF activation is observed in most cancers and contributes to the establishment of immunosuppression in these regions (141, 142). Previous studies have shown that TME contains many types of immunosuppressive cells such as TAMs, MDSCs, and Tregs, all of which are affected by hypoxic stress, particularly HIFs (143).

Because TAMs represent a crucial factor in the immunosuppressive TME, accumulating evidence has indicated that HIF plays a prominent role in the attraction and activation of TAMs. In breast cancer, hypoxia upregulates the expression of CSF-1 via mediation of CCL5/CCR5, which induces the recruitment of TAMs and MDSCs from the peripheral blood to tumor sites and converts them to the suppressive phenotype. Additionally, hypoxic tumor regions stabilize HIF-1α, which in turn upregulates CXCR4 to further increase the chemotactic responsiveness of TAMs and MDSCs (144). Analysis of the link between the innate immune hypoxic response and tumor progression has also shown that activated myeloid HIF-1α regulates arginase and inducible NO synthase expression to induce T-cell suppression in the hypoxic microenvironment, whereas targeted deletion of HIF-1α in TAMs inhibites arginase production and blocks tumor progression (145). Notably, many researchers have postulated that there is an underlying relationship between hypoxia and Tregs (142, 146). Hypoxia promotes immunosuppression by activating the STAT3 pathway via phosphorylation (147), and activated STAT3 in Tregs further inhibits CD8^+^ T-cell differentiation and DC maturation by modulating the expression of FOXP3, TGF-β, and IL-10 (148, 149). Moreover, overexpression of PD-1 on the surface of suppressive cells has been observed in proliferating cancer cells exposed to hypoxia (150). Immunosuppressive factors induced by hypoxia and STAT3 together promote a tumorigenic microenvironment through apoptosis of effector T cells and induction of Tregs.

**Figure 3:**
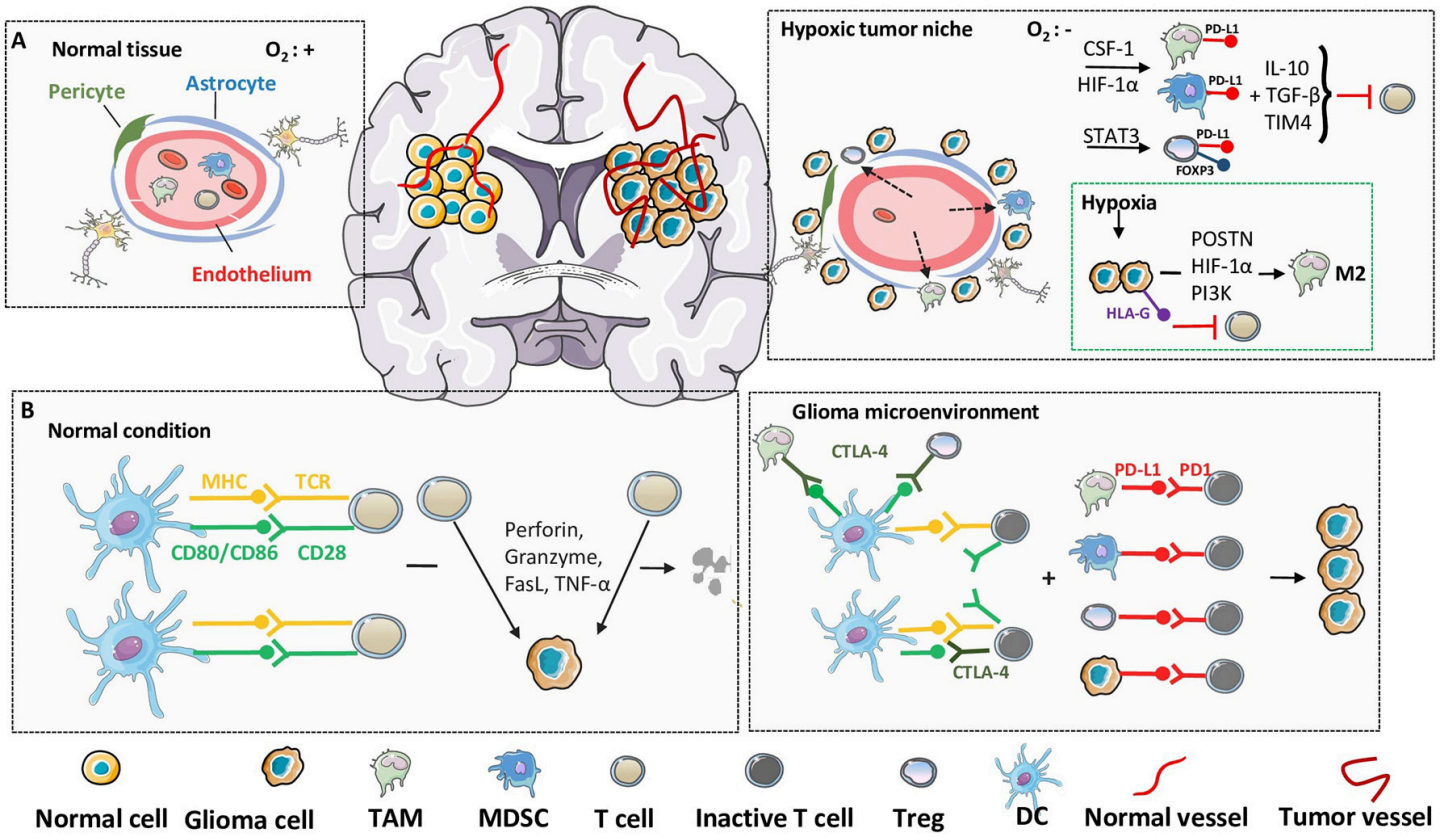
Hypoxia and immune checkpoint in glioma microenvironment. In immunosuppressive glioma microenvironment, despite the direct molecular and cellular mechanisms, the environmental factors, such as hypoxia and immune checkpoint, also play critical roles in the failure of immunosurveillance. **(A)** Compared with normal tissues, inner-glioma environment is always recognized as hypoxic and sufficient in immune cell infiltrations, due to the abnormalities in blood vessel morphology and impairing of BBB integrity. Upregulation of hypoxia-related factors, such as HIF-1α, cooperate with specific immune cells from leaky BBB to establish an immunosuppressive microenvironment and suppress the function of cytolytic T cells. **(B)** Under physiological condition, T cells can be activated through the engagement of MHC to TCR, together with the co-stimulatory signals, to recognize and lyse tumor cells. However, in glioma microenvironment, the elevated expression of CTLA-4 in immunosuppressive cells act as a competitive antagonist of the secondary activation signal, and further lead to the silencing of T cells. Furthermore, induced high expression of PD-1 on T cell surface, as well as PD-L1 on suppressive cells and tumor cells, lead to the anergy and apoptosis of T cells through ligand binding.

Gliomas exhibit a hypoxic environment similar to that of other solid tumors; therefore, the effects of hypoxia on glioma have been investigated in detail (Figure 3). In a hypoxic environment, glioma cells show increased expression of periostin, which promotes the recruitment and migration of TAMs (151). Intriguingly, after recruitment of macrophages to hypoxia areas, macrophage migration is impaired, which could explain why TAMs appear to preferentially accumulate in hypoxic areas (78, 152). Trapped in hypoxic conditions, TAMs are easily converted into specific immunosuppressive M2 phenotype through powerful induction from HIF-1α via RTK/PI3K pathway (151). Additionally, hypoxia during the development of glioma activates HLA-G, an immune checkpoint molecule that contributes to the immune escape of tumor cells (153). In GBM, HIF-1α promotes the recruitment of bone marrow derived CD45^+^ myeloid cells and pericytes by induction of VEGF and SDF-1α, which can enhance vascular remodeling and neovascularization inside tumors (154). Elevated expression of HIF-1α also gives rise to a population of CD133^+^ glioma cancer stem cells, which contribute to hypoxia-induced expansion of Tregs and immunosuppression (155).

### Immune Checkpoints

The augmentation of antitumor immune responses by boosting tumor-specific stimulatory signaling to promote immune-mediated tumor growth arrest has been studied for several years. However, more recent studies have now suggested that a network of suppressive mechanisms may exert a dominant role in the TME, acting in concert to impede immune surveillance and promote tumor development. Based on this perspective, immune checkpoints are thought to play a significant role in generating this immunosuppressive tumor context during the progression of cancer, and the interference with these immune inhibitory checkpoints has attracted attention as a potential strategy for cancer immunotherapy (156, 157). Among the various immune checkpoints being studied, CTLA-4 and PD-1 have emerged as important tools. CTLA-4 (also known as CD152) has been identified as the first negative regulator of T-cell activation and function, and transduces suppressive signals through active antagonism of other costimulatory ligands to CD28 due to its high affinity and ability to exhaust T cells (158, 159). In addition to its direct inhibitory effects, CTLA-4 also enhances Treg-induced immunosuppression. Because CTLA-4 downregulates CD80 and CD86 on antigen-presenting cells, natural Tregs require CTLA-4 to suppress immune responses, which sustain the self-tolerance and immune homeostasis of these cells (160, 161). Similar to CTLA-4, PD-1 is also expressed on the surface of most components of the immune system, such as activated T cells, B cells, NK cells, TAMs, and DCs (162). A number of studies have shown that high PD-1 levels are detected during the activation phase of T cells, compared to the weak expression of PD-1 at the rest (163, 164). Further, elevated expression of PD-1 and its ligand PD-L1 are detectable in various malignant cancers, which connect with the exhaustion of specific T cells (165). The interaction of PD-L1 with PD-1 significantly reduces the production of IFN-γ from activated T cells and leads to T-cell anergy (166). Additionally, PD-L1 also accelerates the differentiation of native CD4^+^ T cells into Tregs and sustains their suppressive phenotype through multiple pathways (167, 168).

Tumor-infiltrating immune cells of glioma patients exhibit significant expression of CTLA-4 (particularly for CD4^+^ effector T cells and Tregs). Since Tregs constitute a major subset of CD4^+^ T cells, elevation in Treg/CD4^+^ T-cell ratio (with pronounced expression of CTLA-4) in TME leads to the inhibition of tumor-reactive T cells, either by direct cell contact or through TGF-β and IL-10, correlating with the disease progression in glioma patients (169, 170). The critical roles of CTLA-4 in regulating immunosuppressive antitumor responses have been illustrated in glioma, which suggest that anti-CTLA-4 treatment will largely enhance CD4^+^ T-cell proliferation and restore the Treg/CD4^+^ ratio to further support the antitumor response (171). Additionally, downregulation of CTLA-4 on peripheral lymphocytes is associated with an improved prognosis in patients with glioma (172). PD-1 is also upregulated in peripheral effector T lymphocytes during glioma disease progression and PD-1 expressing cells extremely high in glioma-infiltrating lymphocytes compared with that of healthy controls (173). Binding of PD-1 with its ligand PD-L1, (expressed at high levels in the immunosuppressive microenvironment due to loss of PTEN [phosphatase and tensin homolog] and activation of the PI3K pathway), generally augments the inhibition of T-cell function and maintains immunological tolerance by impeding the downstream activation pathway and inducing exhaustion (174, 175). Earlier studies reveal that an immunosuppressive state is induced by TAMs or Tregs by the PD-1 axis, which represents crucial cancer-promoting conditions both in neoplastic progression and subsequent immune escape (176). Thus, collectively various studies suggest that blockade of PD-1 signaling may inhibit immune suppression and sustain antitumor immune responses in various cancers, including glioma (Figure 3).

### Immunotherapeutic Strategies Targeting Suppressive TME of Glioma

**Figure 4:**
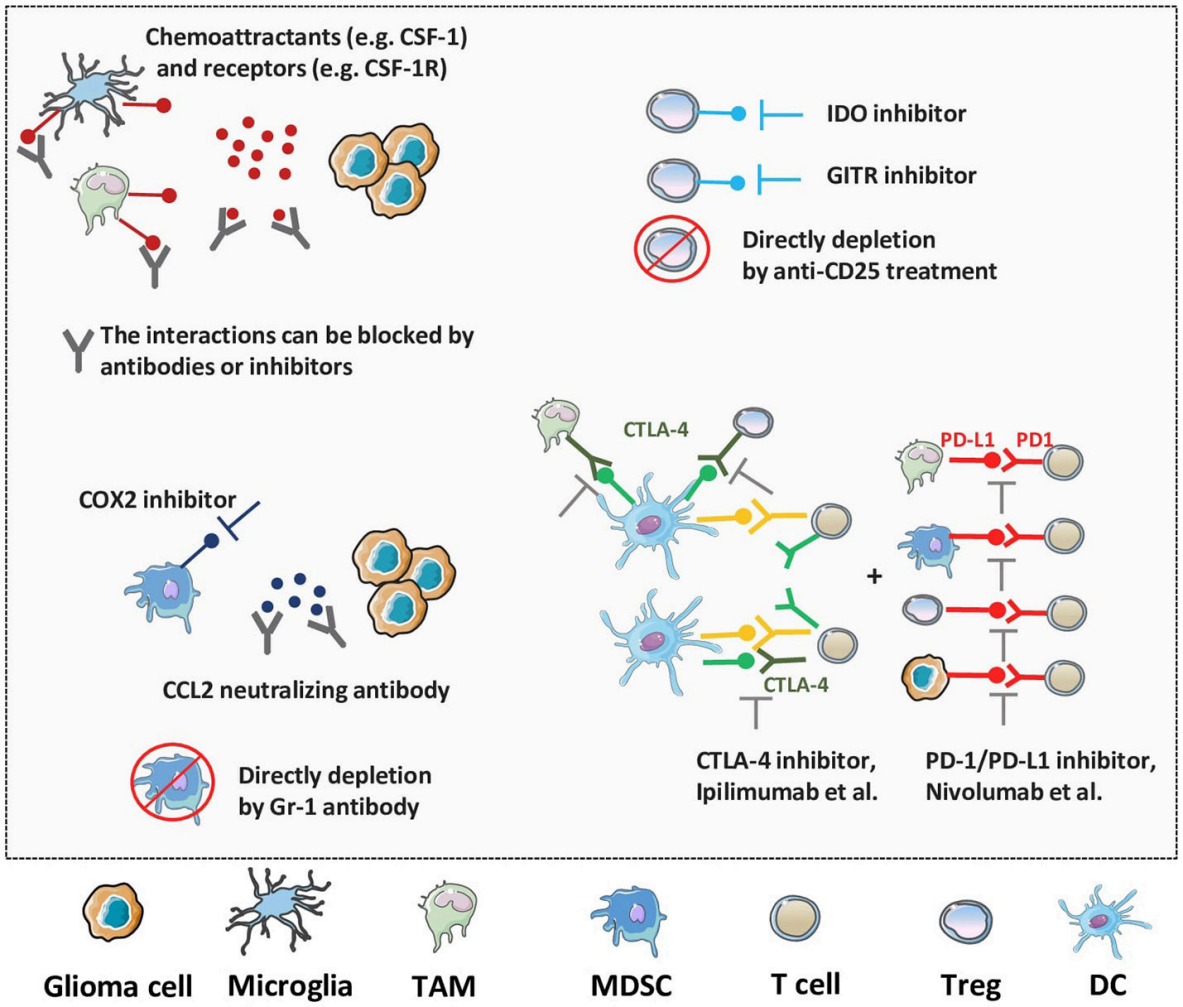
Novel therapeutic strategies against the immunosuppressive TME of glioma. As multiple factors, like TAMs, microglia, MDSCs, Tregs and immune checkpoint molecules, work together to enhance immunosuppression and progression of glioma, various strategies have been generated to target these processes. The blockade of suppressive cell recruitment and migration, impairment of their activities, or direct depletion of these factors will synergistically promote the anti-tumor immune responses and improve the prognosis of glioma patients.

Harnessing the immune system is becoming a novel and most powerful approach to eradicate malignant cells in cancer therapies (177). A range of different immunotherapies targeting the immunosuppressive components of glioma TME are currently being investigated (Figure 4). Since the abundance of TAMs and microglia in glioma exert significant tumor-promoting influence, various therapeutic strategies which modulate their immunosuppressive functions have been investigated (178). TAMs and microglia critically depend on CSF-1R for multiple functions and blockade of CSF-1R has shown therapeutic efficacy in glioma models and patients. In TME, TAMs tend to exhibit tumorigenic M2 phenotype and contribute to glioma development. However, when targeted by CSF1R inhibitors, the TAMs could switch to an anti-tumorigenic M1 phenotype and promote tumor cell death. Administration of PLX3397, a pharmacological inhibitor of CSF-1R which could cross the BBB, reduces the recruitment and invasion of TAMs and microglia *in vivo* (179). Similarly, application of another CSF-1R inhibitor, BLZ945, significantly blocked the progression of established tumors and increased survival in animal models. Interestingly, after treatment with BLZ945, the remaining TAMs show less activated M2 markers with impaired tumor-promoting functions (180). Although CSF1R inhibition is expected to be a promising strategy for glioma treatment, another study suggests the potential drug-resistance mechanisms. The resistance to CSF1R inhibition might be mediated by elevated activities of PI3K and IGF-1/IGF-1R pathways in TME. Therefore, combining CSF-1R blockade with PI3K or IGF-1R inhibition prolongs overall survival in recurrent gliomas (181).

Immunosuppression induced by MDSCs also establishes a huge barrier for immunotherapeutic approaches, and recent therapies aimed at abolishing the activities of these cells have shown to be effective in anti-tumor response. Blockade of cyclooxygenase-2 (COX2) reduces the recruitment and accumulation of MDSCs and leads to the elevated numbers of cytotoxic T cells in glioma TME (182). Moreover, COX2 inhibitor greatly improves the efficacy of other immunotherapies in glioma animal models with significantly prolonged survival time compared with immunotherapy alone (183, 184). It has been demonstrated that CCL2 is crucial in promoting infiltration of MDSCs into tumor sites. Further, systemic administration of CCL2 neutralizing antibody by itself or in combination with temozolomide (TMZ) reduces accumulation of MDSCs and enhances the survival of tumor bearing mice (185). A pre-clinical CAR T cell therapy in mouse models shows that IL13Rα2-CAR T cells not only enhance the abilities of tumor specific T cells but also give rise to a decrease in MDSCs numbers during early stage of treatment which suggests that reducing MDSCs numbers might contribute to additional anti-glioma efficacy (186). For instance, studies have demonstrated that elimination of MDSCs in glioma TME by Gr-1 antibody strongly amplifies the TK/Flt3L gene therapy induced tumor specific T cell response, which results in increased median survival and percentage of long-term survivors (187).

In addition to changing TAMs and MDSCs from an anti- to pro-tumor phenotype, the glioma TME is able to recruit Tregs to tumor site during early stages of tumor progression and inhibit T cell functions. Additional studies are therefore considering Tregs as potential target during immunotherapy (188). Since IDO [indoleamine (2,3)-dioxygenase] expressed by glioma cells is essential for recruitment of Tregs, the use of IDO inhibitors has been shown to decrease the overall accumulation of Tregs and enhance survival in established glioma (189). Another immune checkpoint that is constitutively expressed on Tregs is glucocorticoid-induced TNFR related protein (GITR), and antibodies against GITR, provide another pathway for targeting this suppressive cell type with promising data coming from glioma models (190). Additionally, direct depletion of Tregs through anti-CD25 treatment results in greater accumulation of cytotoxic T cells and infiltration of non-immunosuppressive MDSCs in glioma TME compared to control-treated mice, thereby displaying complete protection against tumor challenge (191).

For glioma immunotherapy, immune checkpoints have received considerable interests due to their profound suppressive potential. Although the list of various immune checkpoints is growing fast, the most commonly studied molecules are PD-1 and CTLA-4 which have already demonstrated promising benefits in patients with glioma in numerous studies (192). The anti-PD-1 antibody nivolumab (inhibitor of immune checkpoint) has advanced farthest clinical development (NCT02550249). The first large phase III trial for GBM initiated in 2014, where nivolumab in recurrent GBM was either used alone or in combination with the anti-CTLA-4 antibody ipilimumab (NCT02017717). Further, pidilizumab, another PD-1 inhibitor, is also being tested for diffuse intrinsic pontine glioma and recurrent GBM (NCT01952769). Moreover, addition of immune checkpoint blockade to conventional glioma treatments has been recognized to enhance the therapeutic efficacy and lead to ample benefits (193). In addition, synergy between checkpoint inhibitors and radio-therapy has been observed in preclinical studies with improved outcomes and decreased toxicity and side effects (194, 195). In another phase III clinical trial (NCT02617589), investigators are exploring nivolumab as an alternative to TMZ, each in combination with radiotherapy, in patients with newly diagnosed MGMT-unmethylated tumors. Similarly, combination of checkpoint inhibition with chemotherapy has also resulted in a favorable outcome (196). While these synergistic combinations have been proved as effective in enhancing antitumor immune responses, ongoing clinical studies might reveal their benefit and complement them as a standard therapeutic strategy in treating the suppressive TME of glioma.

## Conclusion

The studies targeting stem cells and microenvironment of gliomas in recent years have shown that the progression of gliomas critically relies on the functions of these two components. Various components within the tumor bed such as GSCs, different types of immune cells, cytokines, and other molecules, in coordination with each other, contribute to a more supportive TME which promotes glioma cell growth and metastasis. Further investigation of GSCs and the immunosuppressive phenotype caused by the TME will provide opportunities to not only achieve a more comprehensive understanding of tumor biology but also develop specific medical therapies to target the weaknesses underlying tumor development and attack tumor cells in more effective ways. For example, various preclinical studies have clearly demonstrated that combination immunotherapies such as vaccines, Treg depletion, or immune checkpoint blockade, together with chemotherapy have more profound outcomes compared to conventional chemotherapy alone. However, in addition to current advances in immunotherapy and glioma research, additional studies are needed to determine the distinct biological processes and immunosuppressive landscape of various subtypes of glioma to further establish more advanced and personalized treatment strategies.

## Author Contributions

QM, WL, CX, JC, ML, HW, QL, and R-FW wrote the manuscript and provided final approval for the version to be published. QL and R-FW provided the supervision of the entire work.

### Conflict of Interest Statement

The authors declare that the research was conducted in the absence of any commercial or financial relationships that could be construed as a potential conflict of interest.
